# Advancing Behavioral Health Integration in the Program of All-Inclusive Care for the Elderly (PACE)

**DOI:** 10.1093/geroni/igaf122.2257

**Published:** 2025-12-31

**Authors:** Alexa Fleet, Liane Wardlow, Daniel Shalev, Michelle Moses-Eisenstein, William McGuire, Kristen Hartlieb, Harold Pincus

**Affiliations:** University of Massachusetts, Boston, Massachusetts, United States; West Health Policy Institute, San Diego, California, United States; Weill Cornell Medicine, New York, New York, United States; New York State Psychiatric Institute, New York, New York, United States; New York State Office of Mental Health, New York, New York, United States; West Health Policy Institute, San Diego, California, United States; Columbia University, New York, New York, United States

## Abstract

Older adults enrolled in the Program of All-Inclusive Care for the Elderly (PACE) face significant barriers to accessing behavioral health (BH) services despite high rates of BH comorbidities (60%). Integrating BH into PACE programs is critical to achieving truly comprehensive, person-centered care. This poster introduces a conceptual framework for Behavioral Health Integration in PACE (BHI-PACE) and presents current BH practices and challenges within PACE Organizations (POs) from a nationwide assessment. A mixed-methods sequential explanatory approach was used to assess BHI across POs. This study employed (1) a national survey distributed to 148 POs (38 responses, representing 119 PACE sites and 25,806 participants) and (2) semi-structured interviews with 15 POs (representing 58 sites and 10,029 participants). The BHI-PACE framework comprises four domains: (1) Person/Family-Oriented Care, (2) Clinical Functions, (3) Workforce to Support Clinical Functions, and (4) Structures to Support Clinical Functions. Key findings highlight that 60% of PACE participants experience BH comorbidities, with depression (92.1%), behavioral and psychological symptoms of dementia (57.9%), and anxiety disorders (55.3%) as the most common referral reasons. Workforce shortages, insufficient funding structures, and limited integration of BH specialists into POs were identified as primary barriers. However, successful integration strategies include the use of telemedicine, interdisciplinary team collaboration, and formalized external partnerships. This work contributes to both theory and practice by presenting a replicable framework for BH integration in complex care settings for older adults. It informs future policy initiatives, workforce development strategies, and implementation research aimed at improving BH outcomes in aging populations.

